# Editorial: Reinforcement feedback in motor learning: neural underpinnings of skill refinement

**DOI:** 10.3389/fnbeh.2025.1589738

**Published:** 2025-03-31

**Authors:** Christopher M. Hill, Vincent Koppelmans, Mario Manto

**Affiliations:** ^1^School of Kinesiology, Louisiana State University, Baton Rouge, LA, United States; ^2^Department of Psychiatry and Huntsman Mental Health Institute, University of Utah, Salt Lake City, UT, United States; ^3^Service de Neurologie, CHU-Charleroi, Charleroi, Belgium; ^4^Department of Neurosciences, University of Mons, Mons, Belgium

**Keywords:** learning, reinforcement, reward, motor skills, decision making

Making decisions is a critical aspect of human behavior. Reinforcement learning has been investigated in decision-making experiments with the goal of deciphering learning and improve our understanding of how humans make decisions in daily life and in natural environments (Schultz, 2015; Wise et al., 2024). Reinforcement learning of motor skills designates the complex ability of learning from past outcomes with the aim of optimizing rewards, representing a major feature of acquisition of new motor skills (Vassiliadis et al., 2024). Performances adjustments are dictated by reward-prediction errors. Reinforcement feedback promotes motor learning by enhancing retention (Huang et al., 2011).

Anatomically, the brain structures playing a key role in reinforcement learning include the midbrain, striatum, prefrontal cortex and motor cortex (Vassiliadis et al., 2024; Haber, 2016). Cerebellar circuits are also involved through reward mechanisms via the striatum, ventral tegmental area (VTA) and prefrontal cortex (Manto et al., 2024). Physiologically, striatal gamma activity appears critical to reinforce learning of fine skills in human, as shown recently in particular using the technique of transcranial temporal interference stimulation (Vassiliadis et al., 2024). In case of error-based learning, learning is mainly dependent on cerebello-cortical pathways on the basis of forward models involved in the predictions of the impact of future behavior (McNamee and Wolpert, 2019; Manto et al., 2024).

This Research Topic gathers contributions aiming to unravel novel facets of reinforcement learning research. So far, reward prediction errors have been assessed by measuring the reward positivity, an event-related potential extracted from EEG recordings, with a positive deflection at the level of fronto-central areas typically at FCz (Krigolson, 2018). Bacelar et al. show a non-linear relationship between learners' feedback-evoked brain EEG activity and trial accuracy. Interestingly, learners with high performances are more sensitive to violations in reward expectations. Low-performing participants show performance expectations that are uncertain, being unable to differentiate good performances.

What are the interactions between biomechanical control, tactile feedback, and cognitive processing in motor skill acquisition? To answer this question, Cienfuegos et al. studied a complex bimanual task using an original maze game. Participants were asked to move a rolling sphere, employing novel tactile sensors. The authors have introduced cognitive primitives. Good performers showed more efficient navigation, using better motion strategies and improved motor control. They also exhibited a more detailed cognitive representation of the task post-practice. The results highlight the need to consider learning as a set of sophisticated interactions between cognitive events and motor actions.

Dong et al. investigated the effects of football juggling on executive functioning (EF) and functional connectivity in participants aged 17–19 who were randomly assigned to the 70 sessions of juggling (J+; *n* = 38) or no juggling (J–; *n* = 32). The EF components that were assessed pre- and post-juggling were inhibition, working memory, and shifting. Functional MRI with ROI-to-ROI of 132 brain regions, collected pre- and post-juggling, was used to estimate functional connectivity. Significantly more improvement in inhibition and shifting was observed in J+ than in J- participants. A pre-to-post intervention increase in functional connectivity was observed in frontal, temporal and cerebellar regions in the J+ relatively to J– participants. Connectivity between the right superior temporal gyrus and left cerebellum correlated with changes in shifting. These results highlight the neural underpinnings of the association between skill learning and cognitive functioning.

Yin et al. investigated the effects of reinforcement feedback on real-world motor skill learning using a ping-pong ball bouncing task. Participants (*n* = 48) trained for 3 days under reward, punishment, or neutral conditions. Learning, retention, and transfer were assessed pre- and post-training. Punishment enhanced early learning but impaired long-term memory, while reward facilitated late learning and improved short-term memory. Both reinforcement types interfered with long-term memory gains, and effects transferred to the untrained hand. These findings suggest that reward and punishment engage distinct learning processes and neural mechanisms, with implications for motor skill training and rehabilitation.

Hill et al. observed how reinforcement feedback (reward and punishment) modulated locomotor learning behavior. Participants (*n* = 33) learned a new knee flexion pattern during walking, using either reward, punishment, or supervised visual feedback. They observed learning, retention, and savings across these three feedback groups. They found supervised feedback (i.e., enhanced visual feedback) promoted learning and retention more than either reinforcement group. Suggesting that reinforcement may not benefit locomotor adaptation in similar manner seen in upper extremity adaptation. Hence, the effects of reinforcement are task dependent and may even impair certain types of learning.

Together, these studies highlight the complexity of reinforcement learning and the need to pursue original experiments to improve our understanding of relevant goal-directed behavior. Novel paradigms oriented toward naturalistic approaches are needed, as the natural world is highly complex and noisy, rendering decision processes as efficient as possible (Wise et al., 2024). Furthermore, with the advent of novel non-invasive stimulation techniques of the brain, particularly those that can target basal ganglia structures (Riis et al., 2024), it can be speculated that selective modulation of brain regions or networks implicated in reward and reinforcement learning will be applied increasingly frequently (Vassiliadis et al., 2024).
